# Psychosocial functioning in the balance between autism and psychosis: evidence from three populations

**DOI:** 10.1038/s41380-022-01543-5

**Published:** 2022-04-14

**Authors:** Ahmad Abu-Akel, Stephen J. Wood, Rachel Upthegrove, Katharine Chisholm, Ashleigh Lin, Peter C. Hansen, Steven M. Gillespie, Ian A. Apperly, Christiane Montag

**Affiliations:** 1 Institute of Psychology, University of Lausanne, 1015 Lausanne, Switzerland; 2 School of Psychological Sciences, University of Haifa, 31905 Haifa, Israel; 3 School of Psychology, University of Birmingham, Edgbaston, Birmingham, B15 2TT UK; 4 Centre for Youth Mental Health, University of Melbourne, 35 Poplar Rd, Parkville, VIC 3052 Australia; 5 Orygen, Parkville, VIC 3052 Australia; 6 Institute of Clinical Sciences, College of Medical and Dental Science, University of Birmingham, Edgbaston, Birmingham, B15 2TT UK; 7 Forward Thinking Birmingham and Birmingham and Solihull Mental Health Foundation Trust, 1 Printing House Street, Birmingham, B4 6DF UK; 8 Department of Psychology, Aston University, Birmingham, B4 7ET UK; 9 Telethon Kids Institute, The University of Western Australia, 15 Hospital Avenue, Perth, WA 6009 Australia; 10 Department of Primary Care and Mental Health, Institute of Population Health, University of Liverpool, Liverpool, L69 3GB UK; 11 Charité University Medicine Berlin (Charité Universitätsmedizin Berlin), Department of Psychiatry and Psychotherapy, Campus Mitte, Charitéplatz 1, 10117 Berlin, Germany

**Keywords:** Schizophrenia, Genetics

## Abstract

Functional impairment is a core feature of both autism and schizophrenia spectrum disorders. While diagnostically independent, they can co-occur in the same individual at both the trait and diagnostic levels. The effect of such co-occurrence is hypothesized to worsen functional impairment. The diametric model, however, suggests that the disorders are etiologically and phenotypically diametrical, representing the extreme of a unidimensional continuum of cognition and behavior. A central prediction of this model is that functional impairment would be attenuated in individuals with mixed symptom expressions or genetic liability to both disorders. We tested this hypothesis in two clinical populations and one healthy population. In individuals with chronic schizophrenia and in individuals with first episode psychosis we evaluated the combined effect of autistic traits and positive psychotic symptoms on psychosocial functioning. In healthy carriers of alleles of copy number variants (CNVs) that confer risk for both autism and schizophrenia, we also evaluated whether variation in psychosocial functioning depended on the combined risk conferred by each CNV. Relative to individuals with biased symptom/CNV risk profiles, results show that functional impairments are attenuated in individuals with relatively equal levels of positive symptoms and autistic traits—and specifically stereotypic behaviors—, and in carriers of CNVs with relatively equal risks for either disorder. However, the pattern of effects along the “balance axis” varied across the groups, with this attenuation being generally less pronounced in individuals with high-high symptom/risk profile in the schizophrenia and CNV groups, and relatively similar for low-low and high-high individuals in the first episode psychosis group. Lower levels of functional impairments in individuals with “balanced” symptom profile or genetic risks would suggest compensation across mechanisms associated with autism and schizophrenia. CNVs that confer equal risks for both disorders may provide an entry point for investigations into such compensatory mechanisms. The co-assessment of autism and schizophrenia may contribute to personalized prognosis and stratification strategies.

## Introduction

Functional impairment is a core feature of both autism and schizophrenia spectrum disorders (ASD and SSD, respectively) [1], which combined affect approximately 2.5% of individuals during the course of their lifetime [2, 3]. ASD is defined by impairments in social communication and social interactions, and by repetitive behavior and restricted interests and activities. Individuals with ASD also have strong systemizing tendencies, i.e., proclivity to navigate rule-based systems [4]. SSD is associated with the presence of core symptoms that have been classified along negative (e.g., flat or blunted affect, asociality) and positive (e.g., delusions and hallucinations) dimensions, as well as cognitive disorganization. The phenotypic expressions of both disorders are thought to range from attenuated subclinical levels in the general population to severe clinical levels [5–7]. Moreover, while diagnostically independent, the disorders share several clinical and risk factors, and co-occur at rates that exceed estimates within the general population [8–10]. For example, it has been found that ASD occurs, on average, in 24% of people with SSD [8], and in 5% of people with first episode psychosis (FEP) [11]. However, the nature of their association within the individual and how such co-occurrence might impact functional outcomes are unclear. The diametric model [12] posits that ASD and psychosis spectrum disorders—and specifically SSD—are etiologically and phenotypically diametrical, representing the extreme of a unidimensional continuum of cognition and behavior, deviating in opposite directions from typical performance (see also [13]). A central prediction of this model is that functional impairments would be attenuated in individuals with mixed symptom expressions or genetic liability of both disorders. Across three populations, we aim to demonstrate converging evidence in support of this prediction. The examination of the joint effect of ASD and SSD symptoms/genetic risks on functioning is consistent with the need for more transdiagnostic research [14], the use of the Research Domain Criteria framework [15], and the prioritization of improving functional outcome [16].

**Fig. 1:**
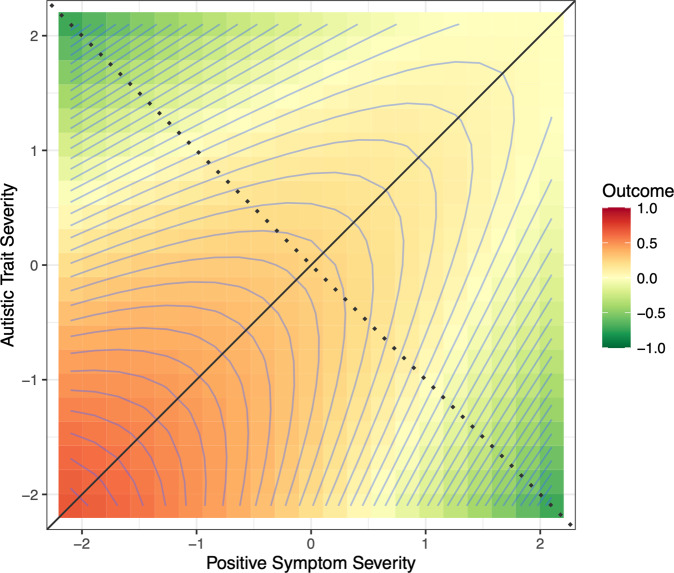
A contour response-surface plot depicting the *hypothesized* association of the co-expression of autistic traits and positive symptoms (or risks) with functional outcome according to the *revised* diametric model. The balance axis (solid diagonal line) represents equal expressions of autistic traits and positive symptoms (or risks). The bias axis (dotted diagonal line) is orthogonal to the balance axis and represents the relative and progressive dominance of autistic traits and/or positive symptoms (or risks), deviating from the center (0, 0) in opposite directions. The contour lines represent functional outcome, ranging from low functional outcome (green) to high functional outcome (red). Along the bias axis, individuals with either elevated autistic traits or positive symptoms (or risks) are equally dysfunctional. Relative to individuals with biased expressions/risks, better functional outcome is expected for individuals with equal expressions/risks. Along the balance axis, functioning, however, is expected to diminish in individuals with high co-expressions of symptoms/risks.

According to the diametric model, ASD is associated with under-developed social cognition and hyper-developed mechanistic cognition, while SSD (and particularly the positive type, e.g., paranoid schizophrenia) with aberrant hyper-developed social cognition and under-developed mechanistic cognition [12]. These abilities represent putative manifestations of evolutionary-genetic tradeoffs of paternally and maternally expressed genes (i.e., genomic imprinting) [17, 18]. A chief support of this model is the presence of pleiotropic rare Copy Number Variants (CNVs)—sites of natural variation in the number of copies of a particular sequence of genomic DNA—which suggests that the disorders are predisposed by a reciprocal biological mechanism [19, 20]. One way of conceptualizing such a tradeoff relationship between ASD and SSD is by estimating the relative expression (or bias) of ASD traits and SSD positive symptoms. We contrast ASD traits and SSD positive symptoms, since only positive symptoms of SSD have been shown to have diametric relations with ASD traits [21, 22]. In healthy individuals, we have shown that the bias score—operationalized as the standardized difference between autistic traits and positive psychotic experiences— follows a curvilinear relationship and is sensitive to variations of socio-cognitive phenotypes in a dose-dependent manner [23], as well as to genotypic variations of genetic risk factors associated with ASD and SSD [24]. Importantly, however, the current conceptualization of the diametric model implies a single ‘tipping point’ along the ASD-SSD axis, beyond which an individual has either an ASD or SSD—and as such rules out ASD-SSD comorbidity unless by chance. This is problematic in light of the recognition that ASD and SSD can be comorbid under some conceptualizations [1, 8–10], and the evidence that some genetic factors may increase risk for both disorders [25–27], barring mistakes in diagnosis. In addition, recent evidence suggests that functional benefits of co-occurring ASD traits and SSD positive symptoms may be delimited by symptom severity [28], which suggests that the degree to which functional impairments may be attenuated in people with mixed symptom expression may be conditional on absolute symptom severity. As such, the bias score may not fully capture outcome variations in people with various levels of symptom co-occurrence. To address these limitations, we propose a revision of the diametric model that allows, within a 3-dimensional framework, for independent variation in ASD and SSD, while recognizing that the relative levels of each contribute to outcomes (see Fig. 1 for details).

This allows us to make a broad set of predictions about the pattern of effects one might see across different populations and different measures of severity or risk for ASD and SSD. To operationalize these predictions in a measurement approach that allows effects associated with the relative level of ASD and SSD to be separated from effects of the absolute level of either disorder, we examine in a series of regression analyses if the effect of one disorder on functioning interactively depended on the relative expression of the other. We complemented these analyses with response surface methodology (RSM) [29], which allowed us to identify the patten of association of different co-occurrence profiles with functional outcome (see Methods). We leverage three existing datasets that had never been analyzed in this way before, and which contained measures that enabled us to test our hypotheses in the domain of psychosocial functioning in: (1) individuals with chronic schizophrenia, (2) individuals with FEP, and (3) healthy carriers of alleles of CNVs conferring risk for both ASD and SSD. Because ASD is made up of distinct phenotypic subdomains—namely, communication, social interaction, and stereotypic behavior—that are dissociable in terms of how they are genetically influenced [30, 31], we also explored if the association between autistic traits and positive symptoms with functioning might be driven by a specific subdomain of ASD. We predicted that functional impairments would be (1) most pronounced in individuals with dominant autistic traits or dominant positive symptoms, and (2) most pronounced in individuals with genetic factors that confer greater risk for ASD relative to SSD (and vice versa). In addition, and relative to individuals with biased symptom/genetic risk profile, we (3) predicted that functional impairments would be attenuated in individuals with relatively equal symptom expressions or genetic risks for ASD and SSD. Furthermore, our analysis also (4) explored if symptom/risk severity would moderate the interaction of ASD and SSD on psychosocial functioning, and specifically if this ‘balanced’ effect diminishes with increasing symptom severity (cf. Figure 1). Although we made no a priori specific predictions about these effects, earlier work has shown that functional benefits of co-occurring ASD traits and SSD positive symptoms in individuals with chronic schizophrenia were not observed in the symptomatically more severe group [28]. The examination of our hypotheses in FEP and chronic schizophrenia allows us to examine if the hypothesized balance effect persists with increasing illness severity. The examination of our hypotheses in healthy CNV carriers allows us to examine if this effect might be linked to ASD and SSD associated genetic risks, and which is not necessarily tied to the manifestation of ASD or SSD symptoms, as well as without the confounding effects of medication or illness duration.

## Methods

Data were obtained from three existing independent datasets: (1) individuals with chronic schizophrenia, (2) individuals with FEP, and (3) healthy carriers of CNVs conferring risk for both ASD and SSD (see description below). Studies were approved by the respective Research Ethics Committee of Charité Universitätsmedizin Berlin (for the schizophrenia group) and the UK Health Research Authority’s National Research Ethics Service (for the FEP group). Written informed consent was obtained from each participant. Ethical approval was not needed for analyses on the healthy CNV carriers group, since data were obtained from published records.

*The chronic schizophrenia group* consisted of 299 individuals, meeting the International Classification of Diseases (ICD-10 [32]) criteria for schizophrenia, F20-F20.9 (for sample characteristics see Supplementary Table S1). Functioning was assessed with the Global Assessment of Functioning (GAF) [33], an interviewer-rated assessment that covers social and occupational functioning. In a subset of this group (N = 120), metacognitive abilities—the capacity to think about thinking—were assessed with the interviewer-rated Metacognition Assessment Scale Abbreviated (MAS-A) [34]. This permitted the examination of our hypothesis with respect to metacognitive abilities as well, which in this subsample, were highly correlated with the GAF (r_ρ_ = 0.60, p < 0.001). These data have not been previously analyzed to address the research questions proposed in this study.

*The FEP group* consisted of 99 individuals presenting in the early stages of psychotic illness, in keeping with the ICD-10 F20-23 and F25-29 criteria (for sample characteristics see Supplementary Table S2). These data were collected as part of a cross-sectional study that examined the clinical significance of autistic traits in people with FEP [35]. Functioning in the FEP group was assessed with the Social and Occupational Functioning Assessment Scale (SOFAS) [36], an interviewer-rated instrument that assesses psychosocial, social, and occupational functioning.

In both the chronic schizophrenia and the FEP groups, positive psychotic symptom severity was assessed with the positive scale of the Positive and Negative Syndrome Scale for Schizophrenia (PANSS) [37], and autistic trait severity was assessed with the PANSS Autism Severity Score (PAUSS) [38]. The PAUSS taps the three symptom subdomains of ASD: (1) “Difficulties in Social Interaction” (the sum of items 1, 3, and 4 from PANSS Negative Scale); (2) “Difficulties in Communication” (the sum of items 5 and 6 from the PANSS Negative Scale); and (3) “Stereotypies/Narrowed Interests” (the sum of item 5 and 15 from the PANSS General Scale, and item 7 from the PANSS Negative Scale). The PAUSS and its subscales is the only validated measure for the assessment of autistic trait severity in SSD (see Supplementary Fig. 1S), and is increasingly recognized as a reliable tool for the assessment of autistic traits and its subdomains in individuals with SSD [39–42], including in individuals with FEP [43]. Importantly, the PAUSS, which was originally validated with the Autism Diagnostic Observation Schedule (ADOS) [44], has been shown to also reflect autistic trait severity of individuals with SSD who were assessed with the Autism Diagnostic Interview- Revised (ADI-R) [45]—a measure that is based on the individual’s early developmental history through a parent/caregiver interview. This suggests that the PAUSS is capturing childhood-onset autistic features rather than current autistic-like features [39, 41]. In this study, Cronbach’s α of the PAUSS was 0.84 in the schizophrenia group and 0.71 in the FEP group. For the PANSS positive, Cronbach’s α was 0.80 in the schizophrenia group and 0.76 in the FEP group.

The data of *the healthy CNV carriers* were obtained from the supplementary material accompanying Stefansson et al. [25], which examined the association of CNVs conferring risk for autism or schizophrenia with fecundity and cognition in healthy carriers. The aspects of the data we used for the purposes of our study consisted of the autism and schizophrenia risk odds ratios (ORs) of 11 CNVs in 142 healthy carriers and their IQ-adjusted impairing effects on functioning. The ORs of risk for one CNV (13q.31.3 Dup, *N* = 5) were not reported, and hence this CNV was not included in our analysis. Thus, our analysis was performed for 10 CNVs in 139 healthy carriers, for which the magnitude of their IQ-adjusted impairing effect on functioning and their ORs of risk for both autism and schizophrenia were fully available. The IQ-adjusted impairing effect of each of the individual CNVs on functioning was estimated by Stefansson et al [25] as the difference in the mean GAF scores of carriers of a particular CNV—which ranged from 5 to 47 carries per CNV—and the mean score of 475 (non-carriers) population controls, conditioned on IQ. The CNVs’ characteristics, ORs, frequency within the analyzed sample, and pooled IQ-adjusted impairing effects on functioning are reported in Supplementary Table S3.

### Statistical analyses

Outcome measures in the chronic schizophrenia and FEP groups were assessed using least-squares regression models in which functioning was estimated with the Z standardized scores of the PANSS positive and PAUSS total scores, and their interaction. Demographic and clinical variables were included in these models as covariates if a variable significantly (*p* < 0.05) correlated with the outcome measure (see Supplementary Tables S1 and S2). Accordingly, gender, verbal IQ, medication dosage and duration of illness were entered as covariates in the chronic schizophrenia group, and gender and education level in the FEP group. We repeated these analyses but with each of the PUASS subdomains in turn, such that the association of positive symptoms and a specific subdomain with functioning was performed while additionally controlling for the other two subdomains. In the healthy CNV carriers group, the outcome measure was the IQ-adjusted CNVs’ impairing effects on functioning, which was estimated with the standardized ORs of risk for autism and schizophrenia and their interaction, while controlling for their ORs of risk for developmental delays, and weighted by the number of carriers of each CNV within the sample. Due to the skewed distribution of the CNVs frequency within the sample, for this analysis, we used Generalized Linear Models. Visualization of the regression results was performed with response surface methodology (RSM) [29], and interaction probes with the Johnson-Neyman method (JNM) [46], using RStudio Version 1.4.1106. RSM enables us to map from the regression models the response surface pattern of the different symptom profiles with level of functioning, and JNM enables to identify the region of significance/insignificance of the interacting variables. Missing values were deleted listwise and so only cases with complete data were analyzed.

## Results

**Fig. 2:**
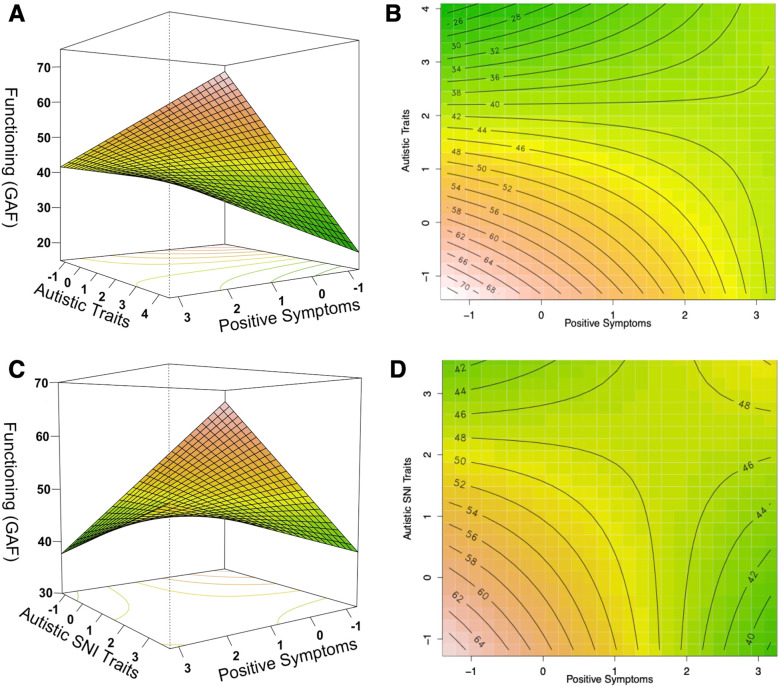
The relationship between autistic traits and positive symptom severity with functioning (GAF) in the chronic schizophrenia group, where *red* corresponds to better functioning and *green* to lower functioning. **A**, **B** show the response surface and corresponding contour plot of the positive interaction between autistic traits and positive symptoms on functioning, with sex, verbal IQ, duration of illness and medication dosage as covariates. **C**, **D** show the response surface and corresponding contour plot of the positive interaction between autistic stereotypies/narrowed interests (SNI) traits and positive symptom on functioning with sex, verbal IQ, duration of illness and medication dosage, autistic communication, and autistic social difficulties traits as covariates. The Johnson-Neyman probes of the interactions are visualized in Supplementary Fig. S2.

In the *chronic schizophrenia group*, the analysis yielded a significant model (F(7,249) = 33.90, *p* < 0.001, R^2^_adjusted_ = 0.47), where better functioning was predicted by a positive interaction between positive symptoms and autistic traits (β(SE) = 1.95 (0.64), t = 3.03, *p* < 0.001). We also observed that functioning was positively associated with verbal IQ and negatively with illness duration and medication dosage (see Supplementary Table S4 for details). In exploring if the observed positive interactive effect on functioning might be driven by a specific subdomain of ASD, we found that it was specifically driven by the interaction of positive symptoms with the PAUSS stereotypies/narrowed interests subdomain (F(9,245) = 26.80, p < 0.001, R^2^_adjusted_ = 0.48; β(SE) = 1.85 (0.67), t = 2.78, *p* < 0.001). Here functioning was also positively associated with verbal IQ and negatively with autistic social difficulties (see Supplementary Table S4 for details). Across both models, the Johnson-Neyman probes revealed that the negative association of positive symptoms with functioning was attenuated with increasing autistic traits/PAUSS stereotypies/narrowed interests, and vice versa (see Supplementary Fig. S2). Moreover, the RSM plots (see Fig. 2A, B) show that while functioning was better in individuals with low-low than those with high-high autistic traits and positive symptoms, individuals with relatively equal levels presented, on average, better functioning than biased individuals. A similar pattern, albeit more robustly, was observed for the positive interaction between positive symptoms and the PAUSS stereotypies/narrowed interests (see Fig. 2C, D).

In a subsample of the schizophrenia group (*N* = 120), we repeated these analyses to estimate metacognitive abilities. We obtained similar results (see Supplementary Fig. S3, Table S5), such that better metacognition was predicted by a positive interaction between positive symptoms and autistic traits (β(SE) = 1.09(0.44), t = 2.50, p = 0.014). However, unlike functioning, the interaction was specifically driven by the PAUSS social and communication difficulties subdomains (see Supplementary Fig. S4, Table S5). Notably, the Johnson-Neyman interaction probe revealed that the negative association of social difficulties with metacognition, reverses and becomes significantly positive when the severity of positive symptoms is ≥ 2.36 SD (see Supplementary Fig. S4F).

**Fig. 3:**
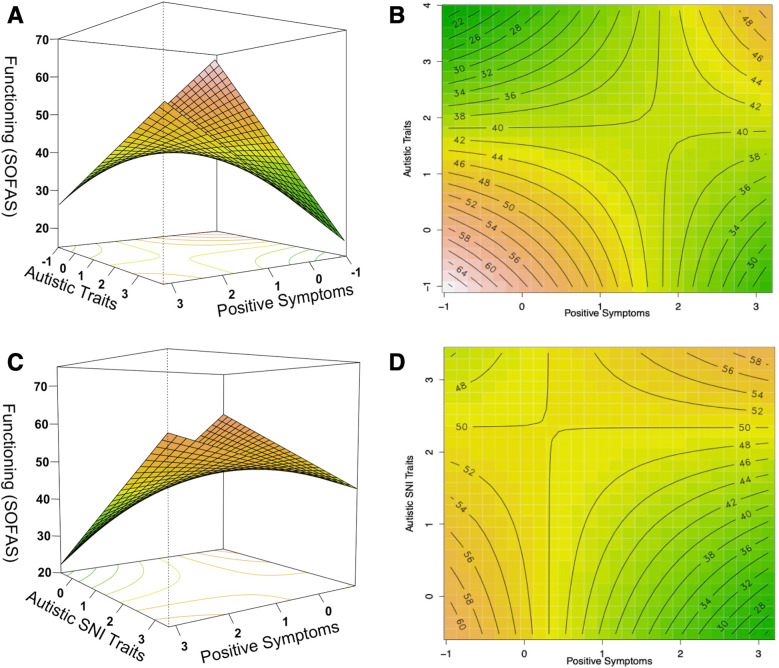
The relationship between autistic traits and positive symptom severity with functioning (SOFAS) in the first episode psychosis group, where *red* corresponds to better functioning and *green* to lower functioning. **A**, **B** show the response surface and corresponding contour plot of the positive interaction between autistic traits and positive symptoms on functioning, with sex and education level as covariates. **C**, **D** show the response surface and corresponding contour plot of the positive interaction between autistic stereotypies/narrowed interests (SNI) traits and positive symptom on functioning, with sex and education level as covariates. The Johnson-Neyman probes of the interactions are visualized in Supplementary Fig. S5.

With respect to the *FEP group*, analysis also yielded a significant model (F(5,89) = 17.43, *p* < 0.001, R^2^_adjusted_ = 0.47), where better functioning was predicted by a positive interaction between positive symptoms and autistic traits (β(SE) = 3.70 (1.73), t = 2.14, p = 0.035), and by sex, where females presented better functioning than males (see Supplementary Table S6 for details). In exploring if the observed interactive effect on functioning might be driven by a specific subdomain of ASD, we found, like in the chronic schizophrenia group, that this positive interactive effect was specifically driven by the interaction of positive symptoms with the PAUSS stereotypies/narrowed interests subdomain (F(7,87) = 13.76, *p* < 0.001, R^2^_adjusted_ = 0.49; β(SE) = 3.28 (1.54), t = 2.12, *p* = 0.036). In this group, functioning was also negatively associated with autistic social difficulties and predicted by sex, where females presented better functioning than males (see Supplementary Table S6). Across both models, the Johnson-Neyman probes revealed that the negative association of positive symptoms with functioning was attenuated with increasing autistic traits/PAUSS stereotypies/narrowed interests, and vice versa (see Supplementary Fig. S5). Notably, this analysis revealed that the negative association of the PAUSS stereotypies/narrowed interests with functioning, reverses and becomes significantly positive when the severity of positive symptoms is ≥2.07 SD (see Supplementary Fig. S5D). Moreover, the RSM plots (Fig. 3) show that functioning was better for individuals with relatively equal autistic traits and positive symptoms than individuals with either elevated autistic traits or positive symptoms. Notably, along the ‘balance axis’, the relationship followed a U-shaped pattern, with low-low and high-high individuals appearing functionally equivalent.

**Fig. 4:**
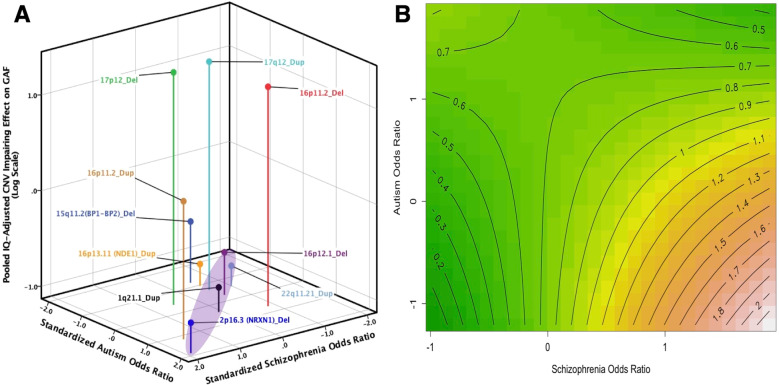
Copy Number Variants (CNVs) conferring risks for autism and schizophrenia and their IQ-adjusted impairing effect on functioning (GAF) in healthy carriers. **A** presents the CNVs’ pooled IQ-adjusted impairing effect on functioning relative to their Z standardized Odds Ratio (OR) of risk for autism and schizophrenia. The CNVs’ impairing effects are presented along a logarithmic scale such that negative values represent smaller effects and positive values represent larger effects. Spikes are visualization aids to help identify the coordinates of the CNVs’ ORs within the 3-D space. The shaded area highlights the balance axis where the Z standardized ORs of risk for autism and schizophrenia are relatively equivalent. **B** is a contour plot of the regression estimating the association of the standardized CNVs’ risk ORs for autism and schizophrenia, and their interaction with the CNVs’ impairing effect on functioning, weighted by their frequency within the sample, and with their OR of risk for developmental delay, as a covariate (see Supplementary Table S7 for details). The contour lines range from low impairing (green) to high impairing (red) effects.

Finally, the model examining the association of the CNVs’ risk for autism and schizophrenia with the magnitude of their IQ-adjusted impairing effects on functioning was significant (Likelihood Ratio χ^2^ = 13.06, *p* = 0.011, adjusted R^2^ = 0.53). The CNVs’ impairing effects on functioning was predicted by a negative interaction between the autism and schizophrenia risk ORs (β(SE) = −0.35(0.12), t = −3.04, *p* = 0.038) (see Table S7 for details). The Johnson-Neyman probe revealed that the positive association of the CNVs’ ORs of risk for schizophrenia with impairment is attenuated with increasing ORs of risk for autism, and vice versa (see Supplementary Fig. S6). Notably, the association of ORs of risk for autism with impairment becomes significantly negative when the ORs of risk for schizophrenia is ≥0.84 SD (see Supplementary Fig. S6B). Moreover, Fig. 4 shows that the impairing effect is, on average, smaller for CNVs conferring relatively equal risks for autism and schizophrenia (e.g., 1q21.1 duplication, 22q11.21 duplication) than CNVs that predispose risk mainly for autism or schizophrenia (e.g., 17p12 deletion, 16p11.2 deletion). Although CNVs with relatively equal smaller ORs of risk are relatively less impairing than CNVs with relatively equal high ORs of risk (Fig. 4B), CNVs with equal high ORs of risk are less impairing, and particularly relative to CNVs with biased risk for schizophrenia.

## Discussion

This study sought to test a central hypothesis of the diametric model of ASD and SSD, postulating attenuated impairments in individuals with equal symptom expressions or genetic risk liability. Results from early and chronic stages of psychotic illness, as well as from healthy carriers of CNVs conferring risk for both autism and schizophrenia provide converging evidence suggesting that functional impairments are attenuated in individuals with relatively equal levels of autistic traits and positive symptoms or associated genetic risk factors, compared to individuals with biased (imbalanced) profiles. This pattern of results also emerges for metacognitive abilities in a subsample of the chronic schizophrenia group. The interaction probes show that the association of the severity of one predictor with functional impairment is arrested, and in some cases even reversed, with increasing severity of the other predictor (significant reversals of effects can be seen in supplementary Figs. S4F, S5D and S6B). These results resonate with recent evidence for better performance outcomes in the domains of attention and social cognition in individuals with comorbid ASD and schizotypal personality disorder, compared to individuals with either disorder alone [47–49], and suggest that relatively balanced symptom expressions or risks can protect against the impairing effects of either disorder.

Moreover, consistent with our proposed model (see Fig. 1), we observed in the schizophrenia (and somewhat in the CNV carriers group) that individuals with low-low presented better functioning than individuals with high-high autistic traits and positive symptoms (or CNV risks) (see Figs. 2 and 4), suggesting that functional benefits may be delimited with increasing co-severity (cf. [28]). However, we additionally observed, and particularly in the FEP group (see Fig. 3), that the association of relatively equal levels of autistic traits and positive symptoms with functioning follows a U-shaped pattern, where low-low and high-high individuals appear to present functional parity. Such reversal of severity effects on functioning may point to a self-stabilizing capacity, which may be underlined by a mechanism that can lead to reversal of pathological mechanism associated with functional impairment. While this proposal is admittedly speculative, it is intriguing to note that such counterintuitive reversal effects have been demonstrated in mice in which *enhancing* related-pathogenic mechanisms led to reversal in depression-related behavior including social interaction deficits [50]. Comparing individuals with low-low, mid-mid, and high-high levels of symptoms severity can lead to important insights in uncovering such mechanism.

Our analyses taking into account the subdomains of autism revealed that reduced functional impairment in both the schizophrenia and the FEP groups is specifically associated with the interaction of stereotypies/narrowed interests subdomain with positive symptoms. This is consistent with findings from two recent studies examining the association of autistic traits in individuals with schizophrenia, where in one, individuals with elevated stereotypic behavior performed better on activities of community living [51], and in the other, stereotypic behavior was predictive of the schizophrenia group with higher functioning [52]. Moreover, this result is also consistent with an earlier study in which performance benefits were observed in individuals with comorbid delusional disorder (formerly paranoia) and obsessive-compulsive disorder (OCD) relative to individuals with either disorder alone [53]. Stereotypic behavior is a main feature that is common to both OCD and ASD. Collectively, these results are consistent with the diametric model [12] and can provide a way of making sense of how proclivity for predictable behaviors and preference for repetitive patterns that characterize ASD—namely, mechanistic/systemizing cognition [4]—can compensate for the dysfunctional hyper-developed mentalistic cognition associated with positive symptoms in SSD [54, 55] (see also [52, 56]). In the context of the interaction between positive symptoms and stereotypic behavior, we reason that such compensation is possible in that stereotypic behavior can facilitate in individuals the ability to establish systematic and predictive relationships among stimuli. In this regard, it is intriguing to note that systematizing the selection of relevant information and environmental cues has been shown to contribute to the success of socio-cognitive remediation in people with schizophrenia [57]. Insofar as stereotypic behaviors are an important aspect of the ASD-SSD relationship, it might be of particular importance to understanding how autistic traits and positive symptoms can confer benefit when co-present, and specifically how non-social ASD-related traits might affect social functioning in schizophrenia. It would be important for future research to also examine the specific relevance of motor versus insistence on sameness or ritualistic types of repetitive behaviors, and to also control for deficits in executive function (e.g., in cognitive flexibility, set shifting and inhibition of pre-potent responses), given their role in the genesis and maintenance of stereotypic/repetitive behavior [30].

Furthermore, we showed that balanced CNVs, namely CNVs that relatively equally predispose individuals to ASD or SSD, generally confer a smaller impairing effect on functioning compared to CNVs that strongly predispose individuals to one disorder or the other. Although this is not without exception (i.e., 17q12 Duplication), this finding advances the notion that functioning might be associated with the relative expression of genes conferring risks for ASD and SSD, and raises the important possibility that a balanced expression of these risk factors can induce an attenuating effect on cognitive and behavioral difficulties associated with these disorders. While obviously these rare CNVs do not account for associated risks in the majority of individuals with ASD or SSD [25, 26], examining the expression of genes and their effects within CNVs that confer risks for both disorders may provide an entry point for investigations into compensatory and protective mechanisms that attenuate functional impairments [25, 26, 58]. In this context, noteworthy is recent evidence showing that duplication carriers of 16p11.2 and 22q11.21—two loci that we have shown to have an attenuated impairing effect on functioning (see Fig. 4)—presented greater levels of stereotypic behaviors than the deletion carriers [59, 60]. This lends further support to the potential central role of stereotypic behavior in understanding how autistic traits and positive symptoms can become adaptive when co-present, and that these CNVs are promising targets in understanding the mechanism underlying their interface.

Currently, the molecular bases that underpin variability in the CNVs’ impairing effects on functioning remain poorly understood. Testing for the effect of risk CNVs for ASD and SSD requires evidence of a molecular mechanism that affects functioning or related abilities/phenotypes in a dose-dependent manner. In this regard, there is encouraging evidence suggesting that a single or small set of dosage sensitive genes harbored within these CNVs might underpin some clinical phenotypes associated with ASD and SSD. For example, the copy number of the CON1 clade subtype of the DUF1220 protein domain—which is mainly encoded by genes of the neuroblastoma breakpoint family (NBPF) on chromosome 1q21—has been shown to contribute to a continuum of severity for both ASD and SSD [21, 61], such that *lower* CON1 copy number are associated with increased positive symptom severity in SSD and *higher* CON1 copy number are associated with increased severity of both negative symptoms in SSD and social and communication symptoms in ASD. The association of the CON1 clade subtype with total gray matter volume and neural proliferation in frontal and parietal cortices has been suggested as a potential mechanism underlying this reciprocal relationship between the expression of positive, on the one hand, and negative/autism symptoms, on the other [21]. Moreover, it has been demonstrated that over- and under-expression of the ASD- and SSD-associated protein CYFIP1—located within the 15q11.2 CNV—regulates, in a dose-dependent manner, the balance between neuronal excitation and inhibition [58]. Consistent with this are findings from a magnetic resonance spectroscopy study showing that autistic and positive schizotypal traits interactively predict the balance between excitatory (glutamate) and inhibitory (GABA + ) neurotransmitter concentrations in the superior temporal cortex—a region involved in social language and functioning [62]—and that excitation/inhibition imbalance is associated with psychosocial deficits [63]. Taken together, these findings make tangible the molecular mechanism underlying the synergistic positive effect of both disorders on functional outcome.

This study has some limitations. First, functioning was assessed using different instruments (GAF in the CNV and schizophrenia groups and SOFAS in the FEP group), whereby, unlike SOFAS, the GAF accounts for functional impairments that are also the result of symptoms. Second, we examined the effect in samples along parts of the psychosis spectrum, but which can be extended to include ultra high-risk individuals for developing psychosis. Future studies should also extend this line of research by examining the effect of co-occurring positive symptoms in samples along the autism spectrum. Third, we utilized the PAUSS—the only validated instrument for the assessment of autistic trait severity in SSD [38]. It is important for the progress of this line of research to develop instruments capable of reliably assessing both autistic traits and positive symptoms transdiagnostically in both ASD and SSD. Fourth, caution should be maintained in interpreting the CNV results due to the overall small sample size, the small number of the CNVs examined, and that not all the CNVs, and particularly 17q12 duplication, support the diametric model. Fifth, it can be argued that the observed effect in the CNV group is due to the *equal risk* CNVs also being lower risk/potency. However, this is unlikely given that 2p16.3 deletion and 16p11.2 duplication, for example, which exert lower functional impairments, also present high ORs of risk for both disorders (see Supplementary Table S3). However, future studies controlling for CNVs’ penetrance are needed to ascertain whether the effect of these CNVs on functioning, and particularly the balanced CNVs, is not due to a lack of penetrance. Finally, future research can extend this aspect of the current work by accounting for potential confounding factors such as whether the CNV is inherited or *de novo*, as well as for potential differences in size and gene content of CNVs at the same locus.

In conclusion, we provide converging evidence suggesting that functional impairments are attenuated in individuals with relatively balanced levels of autistic traits and positive symptom severity or associated genetic risk factors. This suggests that this balance can protect against the deleterious effects of either disorder. The heterogenous pattern of the association of balanced symptoms with functioning, with some depending on symptom severity and some characterized with a U-shaped curve, suggests that functioning in people with a balanced profile might be underpinned by a mechanism that can lead to the arrest or even reversal of pathological mechanism associated with functional impairment. Our results could be a reflection of the variation in the expression of genes conferring risks to either disorder, and raise the possibility that a balanced expression of risk factors results in a functional pattern more similar to individuals in whom these risk factors are absent. Demonstrating that co-occurring ASD and SSD symptoms can have beneficial effects on functioning, underscores the importance of assessing ASD trait severity in individuals with SSD, and challenges current practices viewing ASD and SSD as mutually exclusive disorders. Viewing ASD and SSD as diametric disorders that can co-occur could change the way the field currently examines their impact on behavior, underlying biological systems, and functional outcomes. To have better understanding of these conditions and their heterogeneity, clinicians and researchers will need to routinely assess and account for both disorders. This may be necessary to facilitate the development of individualized and adaptable treatment approaches.

## Supplementary information

Supplemental Material
