# Supplementary material for: Psychosocial functioning in the balance between autism and psychosis: evidence from three populations
Source: Mol Psychiatry. 2022 Apr 14;27(7):2976–84. doi: 10.1038/s41380-022-01543-5 (PMC9205777; doi:10.1038/s41380-022-01543-5)
Supplement: Supplementary file 1 — Supplemental Material [file 41380_2022_1543_MOESM1_ESM.docx]

**Supplementary Material for**

Psychosocial functioning in the balance between autism and psychosis:

Evidence from three populations

**Running title**: Functional benefits of balanced expressions

**Authors**: Ahmad Abu-Akel^1,2*^, Stephen J. Wood^3,4,5^, Rachel Upthegrove^3,6,7^, Katharine Chisholm^3, 8^, Ashleigh Lin^9^, Peter C. Hansen^3^, Steven M. Gillespie^10^, Ian A. Apperly^3^, Christiane Montag^11^

**Affiliations**:

^1^ Institute of Psychology, University of Lausanne, Lausanne 1015, Switzerland

^2^ School of Psychological Sciences, University of Haifa, Haifa 31905, Israel

^3^School of Psychology, University of Birmingham, Edgbaston, Birmingham B15 2TT, United Kingdom

^4^ Centre for Youth Mental Health, University of Melbourne, 35 Poplar Rd, Parkville, VIC 3052, Australia

^5^ Orygen, Parkville, Victoria 3052, Australia

^6^ Institute of Clinical Sciences, College of Medical and Dental Science, University of Birmingham, Edgbaston, Birmingham B15 2TT, United Kingdom

^7^ Forward Thinking Birmingham and Birmingham and Solihull Mental Health Foundation Trust, 1 Printing House Street, Birmingham B4 6DF, United Kingdom

^8^ Department of Psychology, Aston University, Birmingham B4 7ET, United Kingdom

^9^ Telethon Kids Institute, The University of Western Australia, 15 Hospital Avenue, Perth, WA 6009, Australia

^10^ Department of Primary Care and Mental Health, Institute of Population Health, University of Liverpool, Liverpool L69 3GB, UK

^11^ Charité University Medicine Berlin (Charité Universitätsmedizin Berlin), Department of Psychiatry and Psychotherapy, Campus Mitte, Charitéplatz 1, 10117 Berlin, Germany

**Corresponding author:** Ahmad Abu-Akel, Institute of Psychology, Géopolis-Moulin, University of Lausanne, Lausanne 1015, Switzerland. Email: ahmad.abuakel@unil.ch.


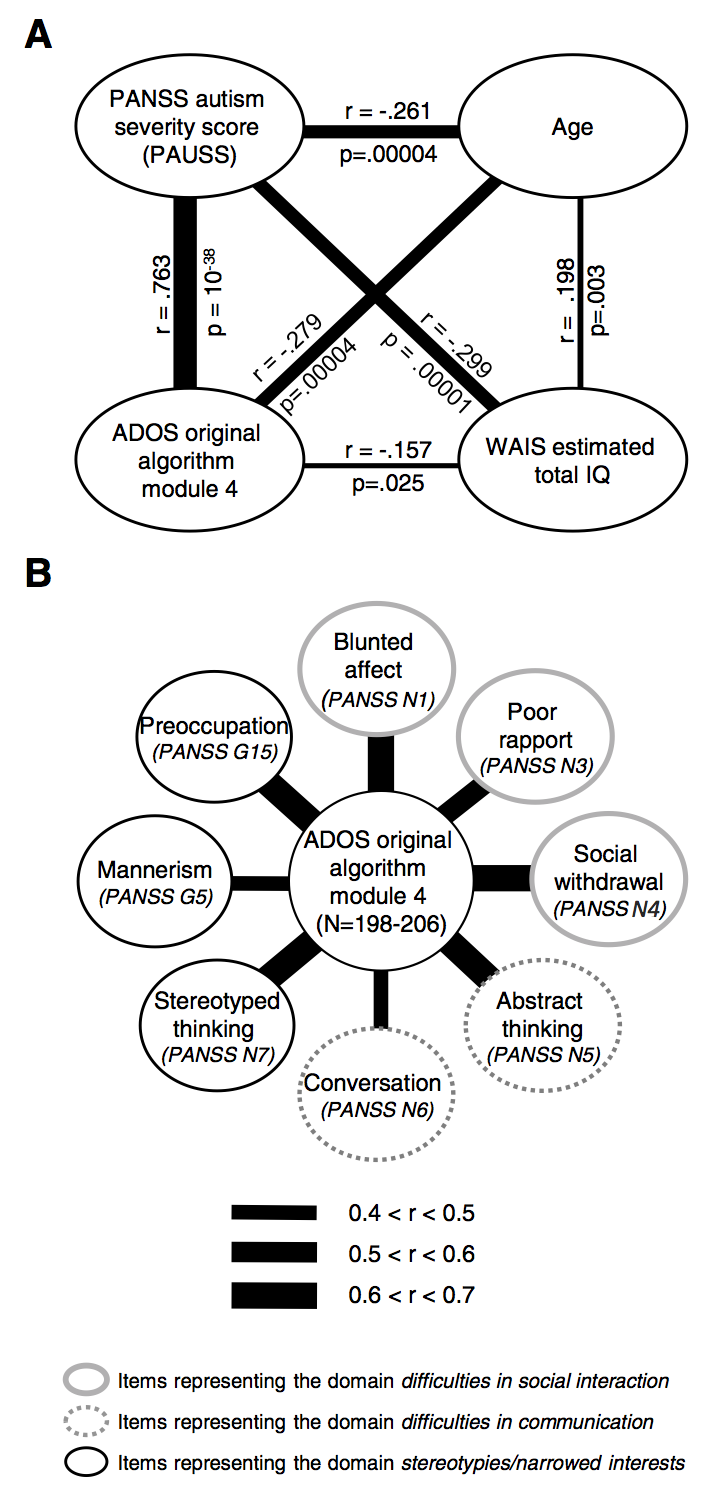


**Figure S1.** Validation of the PANSS Autism Severity Score (PAUSS) in 165 individuals with autism spectrum disorders. Figure shows Spearman rank intercorrelations of individual PAUSS items and the Autism Diagnostic Observation Schedule (ADOS). Figure adapted from Kästner et al (*BMC psychiatry 15:115; 2015*) and reproduced with permission under the terms of the Creative Commons Attribution License.

**Figure S2**. The Johnson-Neyman probe of the positive interaction between autistic traits and positive symptom severity and of the positive interaction between autistic stereotypies/narrowed interests (SNI) traits and positive symptoms on functioning (GAF) in the chronic schizophrenia group. For the positive interaction between autistic traits and positive symptom severity, **A** shows that the negative association of positive symptoms with functioning is significantly attenuated with increasing autistic traits (and vice versa in **B**). These negative associations with functioning cease to be significant when the severity for autistic traits and positive symptoms is respectively ≥ 1.14 SD and 2.02 SD. For the positive interaction between autistic SNI traits and positive symptoms, **C** shows that the negative association of positive symptoms with functioning is significantly attenuated with increasing autistic SNI traits (and vice versa in **D**). These negative associations with functioning cease to be significant when the severity for autistic traits and positive symptoms is respectively ≥ 1.24 SD and 0.72 SD.

**Figure S3**. The relationship between autistic traits and positive symptom severity with metacognitive abilities (MAS-A) in a subset of the chronic schizophrenia group (N = 120). **A** and **B** show the response surface and corresponding contour plot of the positive interaction between autistic traits and positive symptoms on metacognition, with sex and verbal IQ as covariates. The contour lines represent outcome, ranging from low metacognitive abilities (green) to high metacognitive abilities (red). **C** and **D** depict the results of the Johnson-Neyman interaction probe. In **C**, the negative association of positive symptoms with metacognition is significantly attenuated with increasing autistic traits (and vice versa as shown in **D**). These negative associations cease to be significant when the severity for autistic traits and positive symptoms is respectively ≥ 1.35 and 0.65 SD.

**Figure S4**. The association of autistic social and communication difficulties traits and positive symptoms with metacognitive abilities (MAS-A) in a subset of the chronic schizophrenia group (N = 120). **A** and **B** show the response surface and corresponding contour plot of the positive interaction between autistic social difficulties and positive symptoms on metacognition, with autistic stereotypies/narrowed interests, autistic communication difficulties, sex and verbal IQ as covariates. **C** and **D** show the response surface and corresponding contour plot of the positive interaction between autistic communication difficulties and positive symptoms on metacognition, with autistic stereotypies/narrowed interests, autistic social difficulties, sex and verbal IQ as covariates. The contour lines represent outcome, ranging from low metacognitive abilities (green) to high metacognitive abilities (red). **E - H** depict the Johnson-Neyman interaction probe. In **E**, the negative association of positive symptoms with metacognition is significantly attenuated with increasing autistic social difficulties (and vice versa as seen in **F**). These negative associations cease to be significant when the severity for autistic social difficulties and positive symptoms is respectively ≥ 1.33 and 0.29 SD. Notably, the negative association of autistic social difficulties with metacognition reverses and becomes significantly positive when the severity of positive symptoms is ≥ 2.36 SD. In **G**, the negative association of positive symptoms with metacognition is significantly attenuated with increasing autistic communication difficulties (and vice versa as seen in **H**). These negative associations cease to be significant when the severity for autistic communication difficulties and positive symptoms is respectively ≥ 1.13 and 0.45 SD. SocD = Social difficulties; ComD = Communication difficulties; AT = Autistic traits; PS = Positive symptoms.

**Figure S5**. The Johnson-Neyman probe of the positive interaction between autistic traits and positive symptom severity and of the positive interaction between autistic stereotypies/narrowed interests (SNI) traits and positive symptoms on functioning (SOFAS) in the first episode psychosis (FEP) group. For the positive interaction between autistic traits and positive symptom severity, **A** shows that the negative association of positive symptoms with functioning is attenuated with increasing autistic traits (and vice versa as seen in **B**). These negative associations with functioning cease to be significant when the severity for autistic traits and positive symptoms is respectively ≥ 0.78 and 0.77 SD. For the positive interaction between autistic SNI traits and positive symptoms, **C** shows that the negative association of positive symptoms with functioning is attenuated with increasing autistic SNI traits (and vice versa as seen in **D**). The negative association of positive symptoms with functioning ceases to be significant when the severity for autistic SNI traits is ≥ 1.11 SD; the negative association of autistic SNI traits with functioning reverses and becomes significantly positive when the severity of positive symptoms is ≥ 2.07 SD.

**Figure S6**. The Johnson-Neyman probe of the negative interaction between the Copy number variants’ (CNVs) Odd Ratios (ORs) of risk for autism and schizophrenia with their IQ-adjusted impairing effect on functioning (GAF) of healthy carriers. **A** shows that the positive association of the CNVs’ impairing effect with their ORs of risk for schizophrenia is attenuated with increasing ORs of risk for autism, and becomes nonsignificant when the OR is ≥ 0.53 SD. Conversely, **B** shows that the positive association of the CNVs’ impairing effect with their ORs of risk for autism is attenuated with increasing ORs of risk for schizophrenia, and abecomes significantly negative when the OR is ≥ 0.84 SD.

**Table S1**. Characteristics of the chronic schizophrenia group (N = 299)

| **Variable** | **Mean ± SD/ Frequency** | **Spearman’s rho with GAF** |
| --- | --- | --- |
| GAF | 56.40 ± 14.51 | -- |
| Positive symptoms (range) | 15.21 ± 6.25 (7, 37) | -0.54*** |
| PAUSS (range) | 15.38 ± 6.28 (7, 43) | -0.63*** |
| Gender (Male:Female)* | 175:120 | 0.12^✪ ^(Females > Males) |
| Age (years) | 37.91 ± 11.17 | -0.09 |
| Education (years) | 13.98 ± 3.43 | 0.27*** |
| Verbal IQ (WST or MWT-B) | 103.92 ± 13.95 | 0.17** |
| Duration of illness (years) | 11.80 ± 9.17 | -0.17** |
| Medication dosage (WHO 2016 Daily Dose) | 1.33 ± 1.15 | -0.22*** |

GAF = Global Assessment of Functioning; WST = Wortschatztest; MWT-B = Mehrfachwahl-Wortschatz-Intelligenztest

^2^ Spearman’s rho for PAUSS and PANSS positive = 0.56***

* The numbers do not add to 299, due to missing data.

^✪^p = 0.053, * p < 0.05, ** p < 0.01, *** p < 0.001

**Table S2**. Characteristics of the First Episode Psychosis (FEP) group (N = 99)

| **Variable** | | **Mean ± SD /Frequency** | **Spearman’s rho with SOFAS**^2^ |
| --- | --- | --- | --- |
| SOFAS | | 51.95 ± 19.56 | -- |
| Positive symptoms (range)^3^ | | 12.34 ± 5.65 (7, 30) | -.50*** |
| PAUSS (range)^3^ | | 12.94 ± 4.88 (8, 32) | -.50*** |
| Age (years) | | 25.45 ± 5.01 | -0.10 |
| Gender (Male:Female) | | 67:32 | 0.33*** (Females > Males) |
| Medication Status (Yes:No) | | 86:13 | 0.00 |
| Education level^1^ | |  | 0.39*** |
|  | No school certificate or any other qualification | 9 |  |
|  | GCSE/NVQ level 1 or 2 | 39 |  |
|  | A-level/GNVQ/ BTEC/NVQ level 3 | 26 |  |
|  | NVQ level 4 or above | 1 |  |
|  | Higher National Diploma (HND) | 4 |  |
|  | Undergraduate degree | 14 |  |
|  | Postgraduate degree | 3 |  |

^1^ IQ data were not available for this group. We note, however, that education has been shown to be highly correlated (*r*= 0.63) with estimated full scale IQ [Matarazzo, J.D., Herman, D.O. (1984). Relationship of education and IQ in the WAIS—R standardization sample. *Journal of Consulting and Clinical Psychology, 52*(4), 631–634].

^2^ SOFAS = Social and Occupational Functioning Assessment Scale

^3^ Spearman’s rho for PAUSS and PANSS positive = 0.40***

* p < 0.001.

**Table S3.** List of Copy Number Variants (CNVs)^1^, their pooled IQ-adjusted impairing effect on GAF, distribution within the sample of healthy carriers (N = 139)^2^: copy state, chromosome, odds ratio (OR)^3^ of CNVs’ risk for autism (AUT), schizophrenia (SZ), and developmental delay (DD).

| **Locus** | **Copy**  **State** | **Chr.**  **Number** | **OR AUT**  **(Z)** | **OR**  **SZ**  **(Z)** | **OR**  **DD**  **(Z)** | **No. of carriers** | **Pooled**  **IQ-adjusted impairing effect on GAF** |
| --- | --- | --- | --- | --- | --- | --- | --- |
| 1q21.1_Dup | Duplication | 1 | 8.00  (0.39) | 4.20  (0.08) | 4.40  (0.13) | 10 | 0.36 |
| 2p16.3 (NRXN1)_Del | Deletion | 2 | 15.10  (1.87) | 10.70  (1.89) | 10.80  (1.98) | 5 | 0.41 |
| 15q11.2(BP1-BP2)_Del | Deletion | 15 | 0.30  (-1.21) | 2.10  (-0.51) | 1.90  (-0.59) | 47 | 0.57 |
| 16p13.11 (NDE1)_Dup | Duplication | 16 | 1.50  (-0.96) | 2.00  (-0.53) | 2.40  (-0.44) | 22 | 0.27 |
| 16p12.1_Del | Deletion | 16 | 4.70  (-0.29) | 1.80  (-0.59) | 2.80  (-0.33) | 7 | 0.34 |
| 16p11.2_Del | Deletion | 16 | 9.50  (0.71) | 0.90  (-.84) | 9.20  (1.51) | 7 | 0.68 |
| 16p11.2_Dup | Duplication | 16 | 11.80  (1.18) | 9.40  (1.53) | 3.40  (-0.16) | 7 | 0.75 |
| 17p12_Del | Deletion | 17 | 4.00  (-0.44) | 5.70  (0.50) | 0.80  (-0.90) | 6 | 1.11 |
| 17q12_Dup | Duplication | 17 | 2.83  (-0.68) | 2.00  (-0.53) | n/a | 7 | 1.43 |
| 22q11.21_Dup | Duplication | 22 | 3.30  (-0.58) | 0.40  (-0.98) | 3.70 | 21 | 0.29 |

^1^ In supplementary Table 1 of Stefansson et al [*Nature* Jan 16 2014;505(7483):361-366.], data were provided for 26 independent CNV regions. However, we were only able to use 10 CNVs, since the impairing effects of the remaining CNVs on functioning were not computed as they have a low number of CNV carriers (N < 5), or because no odds ratios for autism and/or schizophrenia were provided.

^2^ Data on age and gender distribution were not reported. However, participants’ age ranged from 18 to 65 years.

^3^ The confidence intervals of the ORs were not provided.

**Table S4**. Regression results for functioning in the chronic schizophrenia group

| **Variable** | **Estimate** | **Standard Error** | **t-value** | **p-value** |
| --- | --- | --- | --- | --- |
| *The association of functioning with autistic traits and positive symptom severity*  *F(7,249) = 33.90, p < 2.2x10^16^, R^2^_adjusted_= 0.47* | | | | |
| Positive symptoms | -4.25 | 0.86 | -8.25 | **9.32x10^15^** |
| Autistic traits | -6.72 | 0.82 | -4.96 | **1.28x10^6^** |
| Positive symptoms  x Autistic traits | 1.95 | 0.64 | 3.03 | **2.73x10^3^** |
| Sex: Female | 0.29 | 1.38 | 0.21 | 0.835 |
| Verbal IQ | 0.14 | 0.05 | 2.81 | **0.005** |
| Duration of illness | -0.15 | 0.07 | -2.07 | **0.040** |
| Medication Dosage | -1.25 | 0.61 | -2.03 | **0.043** |
| *The association of functioning with autistic* stereotypies and narrow interest *(SNI) traits and positive symptom severity*  *F(9,245) = 26.80, p < 2.2x10^16^, R^2^_adjusted_= 0.48* | | | | |
| Positive symptoms | -4.49 | 0.88 | -5.12 | **6.10x10^7^** |
| Autistic SNI traits | -3.21 | 0.93 | -3.45 | **6.63x10^4^** |
| Positive symptoms x autistic SNI traits | 1.85 | 0.67 | 2.78 | **5.87x10^3^** |
| Autistic communication difficulties | -1.28 | 0.96 | -1.33 | 0.183 |
| Autistic social difficulties | -3.33 | 0.95 | -3.50 | **5.56x10^4^** |
| Sex: Female | -0.05 | 1.43 | -0.04 | 0.971 |
| Verbal IQ | 0.14 | 0.05 | 2.77 | **5.59x10^3^** |
| Duration of illness | -0.14 | 0.07 | -1.85 | 0.066 |
| Medication Dosage | -1.11 | .62 | -1.79 | 0.074 |

**Table S5**. Regression results for metacognitive abilities (MAS-A) in a subsample of the chronic schizophrenia group (N = 120)

| **Variable** | **Estimate** | **Standard Error** | **t-value** | **p-value** |
| --- | --- | --- | --- | --- |
| *The association of metacognition with autistic traits and positive symptom severity*  *F(5,113) = 26.58, p < 2.2x10^16^, R^2^_adjusted_= 0.52* | | | | |
| Positive symptoms | -2.77 | 0.48 | -5.80 | **6.12x10^8^** |
| Autistic traits | -1.83 | 0.46 | -3.94 | **1.40x10^4^** |
| Positive symptoms  x Autistic traits | 1.09 | 0.44 | 2.50 | **0.014** |
| Sex: Female | 0.74 | 0,70 | 1.07 | 0.288 |
| Verbal IQ | 0.10 | 0.03 | 3.99 | **1.16x10^4^** |
| *The association of metacognition with autistic social difficulties and positive symptom severity*  *F(7,111) = 24.64, p < 2.2x10^16^, R^2^_adjusted_= 0.58* | | | | |
| Positive symptoms | -2.94 | 0.46 | -6.40 | **3.76x10^9^** |
| Autistic social difficulties | -1.29 | 0.44 | -2.94 | **3.94x10^3^** |
| Positive symptoms x Autistic social difficulties | 1.36 | 0.36 | 3.78 | **2.59x10^4^** |
| Autistic communication difficulties | -1.55 | 0.56 | -2.75 | **6.98x10^3^** |
| Autistic stereotypies/narrowed interests | 0.41 | 0.47 | 0.87 | 0.385 |
| Sex: Female | 0.41 | 0.68 | 0.61 | 0.546 |
| Verbal IQ | 0.08 | 0.03 | 3.10 | **2.48x10^3^** |
| *The association of metacognition with autistic communication difficulties and positive symptom severity*  *F(7,111) = 22.77, p < 2.2x10^16^, R^2^_adjusted_= 0.56* | | | | |
| Positive symptoms | -2.56 | 0.45 | -5.65 | **1.28x10^7^** |
| Autistic communication difficulties | -1.71 | 0.58 | -2.95 | **3.90x10^3^** |
| Positive symptoms x Autistic communication difficulties | 1.17 | 0.40 | 2.91 | **4.33x10^3^** |
| Autistic social difficulties | -1.25 | 0.45 | -2.77 | **6.53x10^3^** |
| Autistic stereotypies/narrowed interests | 0.23 | 0.48 | 0.48 | 0.634 |
| Sex: Female | 0.57 | 0.70 | 0.82 | 0.417 |
| Verbal IQ | 0.07 | 0.03 | 2.80 | **6.00x10^3^** |

**Table S6**. Regression results for functioning (SOFAS) in the first episode psychosis (FEP) group (N = 99)

| **Variable** | **Estimate** | **Standard Error** | **t-value** | **p-value** |
| --- | --- | --- | --- | --- |
| *The association of functioning with autistic traits and positive symptom severity.*  *F(5,89) = 17.43, p = 5.38x10^12^, R^2^_adjusted_= 0.47* | | | | |
| Positive symptoms | -6.64 | 1.61 | -4.12 | **8.33x10^5^** |
| Autistic traits | -6.60 | 1.63 | -4.05 | **1.10x10^4^** |
| Positive symptoms x Autistic traits | 3.70 | 1.73 | 2.14 | **0.035** |
| Sex: Female | 11.10 | 3.24 | 3.43 | **9.28x10^4^** |
| Education Level | 1.38 | 0.95 | 1.46 | 0.149 |
| *The association of functioning with autistic stereotypies/narrowed interests traits and positive symptom severity*  *F(7,87) = 13.76, p = 7.26x10^12^, R^2^_adjusted_= 0.49* | | | | |
| Positive symptoms | -7.67 | 1.71 | -4.49 | **2.15x10^5^** |
| Autistic stereotypies/narrowed interests | -1.02 | 1.90 | -0.54 | 0.591 |
| Positive symptoms x Autistic stereotypies/narrowed interests | 3.28 | 1.54 | 2.12 | **0.036** |
| Autistic communication difficulties | -0.20 | 1.94 | -0.10 | 0.917 |
| Autistic social difficulties | -6.41 | 1.80 | -3.56 | **6.03x10^4^** |
| Sex: Female | 13.44 | 3.16 | 4.26 | **5.16x10^5^** |
| Education Level | 1.62 | 0.98 | 1.65 | 0.102 |

**Table S7**. Regression results for the IQ-adjusted CNV impairing effect on functioning (GAF) in the CNV healthy carrier group (N = 139)

| **Variable** | **Estimate** | **Standard Error** | **t-value** | **p-value** |
| --- | --- | --- | --- | --- |
| *The association of the Z standardized odds ratios (ORs) of risk for autism and schizophrenia on the IQ-adjusted CNV impairing effect on functioning, weighted by the frequency of the CNVs within the sample*  *X^2^_df=4_  = 13.06, p = 0.011, R^2^_adjusted_= 0.53* | | | | |
| Risk ORs for schizophrenia | 0.55 | 0.20 | 2.68 | 0.056 |
| Risk ORs for autism | -0.23 | 0.25 | -0.91 | 0.413 |
| Risk ORs for schizophrenia x Risk ORs for autism | -0.35 | 0.12 | -3.04 | **0.038** |
| Risk ORs for Developmental Delay | 0.156 | 0.17 | 0.91 | 0.414 |
